# Dopaminergic neurodegeneration in the substantia nigra is associated with olfactory dysfunction in mice models of Parkinson’s disease

**DOI:** 10.1038/s41420-023-01684-8

**Published:** 2023-10-21

**Authors:** Yu Yuan, Xizhen Ma, Xiaoqing Mi, Le Qu, Meiyu Liang, Mengyu Li, Youcui Wang, Ning Song, Junxia Xie

**Affiliations:** 1 Institute of Brain Science and Disease, School of Basic Medicine, Shandong Provincial Collaborative Innovation Center for Neurodegenerative Disorders, Shandong Provincial Key Laboratory of Pathogenesis and Prevention of Neurological Disorders, Qingdao University, Qingdao, 266071 China; 2 Lingang Laboratory, Shanghai, 200031 China; 3 School of Life Science and Technology, ShanghaiTech University, Shanghai, 201210 China

**Keywords:** Pathogenesis, Diseases

## Abstract

Olfactory dysfunction represents a prodromal stage in Parkinson’s disease (PD). However, the mechanisms underlying hyposmia are not specified yet. In this study, we first observed an early olfactory dysfunction in mice with intragastric rotenone administration, consistent with dopaminergic neurons loss and α-synuclein pathology in the olfactory bulb. However, a much severer olfactory dysfunction was observed without severer pathology in olfactory bulb when the loss of dopaminergic neurons in the substantia nigra occurred. Then, we established the mice models by intrastriatal α-synuclein preformed fibrils injection and demonstrated the performance in the olfactory discrimination test was correlated to the loss of dopaminergic neurons in the substantia nigra, without any changes in the olfactory bulb analyzed by RNA-sequence. In mice with intranasal ferric ammonium citrate administration, we observed olfactory dysfunction when dopaminergic neurodegeneration in substantia nigra occurred and was restored when dopaminergic neurons were rescued. Finally we demonstrated that chemogenetic inhibition of dopaminergic neurons in the substantia nigra was sufficient to cause hyposmia and motor incoordination. Taken together, this study shows a direct relationship between nigral dopaminergic neurodegeneration and olfactory dysfunction in PD models and put forward the understandings that olfactory dysfunction represents the early stage of neurodegeneration in PD progression.

## Introduction

Olfactory dysfunction is commonly considered as a prodromal stage in Parkinson’s disease (PD), affecting 75-90% of the patients [1, 2]. Due to the sensitivity, inexpensivity and feasibility features, olfactory testing has been applied commonly in the initial diagnose of newly diagnosed patients with PD [3]. It was reported that hyposmia increased the risk of dementia in the follow up period of PD patients, suggesting its broader application in the evaluation of PD progression [4]. More recently in a multicenter study, hyposmia was reported in 159/263 (60.5%) idiopathic rapid eye movement sleep behavior disorder subjects, which was much higher than the occurrence of constipation (111/263, 42.2%) [5]. This reinforces the prevalence of olfactory dysfunction in PD diagnosis, even in the preclinical stages of PD. However, the mechanisms underlying hyposmia are not fully specified yet in PD.

Olfactory bulb (OB) is the primary olfactory center in the brain. OB is also described as an early affected area in the Braak Staging, and aggregation of α-synuclein was usually believed to be associated the early non-motor symptom (hyposmia) [6]. It is reasonable to apply α-synuclein aggregates in the OB, or via the olfactory epithelium to investigate α-synuclein spreading and PD pathogenesis in a prion-like manner [7–9]. Similarly, when double-mutant human α-synuclein were overexpressed and aggregated specifically in the OB by an adeno-associated viral vector, the olfactory dysfunction was detected in those mice. The spontaneous activity and odor-evoked firing rates of single mitral/tufted cells were increased, and this hyperactivity might explain the neural circuit mechanisms underlying olfactory dysfunction induced by α-synuclein aggregates [10, 11]. However, in light of the early preclinical onset of olfactory dysfunction in many neurodegenerative diseases, olfactory function is complicated and not be explained only by OB involvements [12]. Even in the normal aged individuals, neither OB tau, Aβ or α-synuclein significantly predict olfactory performance, according to a recent large autopsy study, further suggesting the involvement of other brain regions [13]. Interestingly, functional magnetic resonance imaging analysis revealed that about 0.6% women remains olfaction in the absence of anatomically defined OB [14], raising the possibility that olfactory dysfunction is not necessarily dependent solely on pathological alterations in the OB.

Rotenone is an active ingredient of hundreds of pesticides and pesticides, considered to be one of the environmental neurotoxins implicated in etiopathogenesis of PD. As a naturally inhibitor of mitochondrial complex I, intragastric administration of rotenone is believed to almost reproduce the typical pathological features of PD, that is, accumulated α-synuclein spreading from the enteric nervous system to the central nervous system and the loss of dopaminergic neurons. They also replicate several clinical features, that is, the gastrointestinal and olfactory dysfunction as well as motor incoordination [15–17]. In the present study, we first established rotenone mice models to evaluate olfactory function. An impaired olfactory discrimination was observed early, consistent with α-synuclein pathology in the OB. Unexpectedly, a severer olfactory dysfunction was observed when the loss of dopaminergic neurons in the substantia nigra pars compacta (SNpc) occurred. We then used mice models with intrastriatal α-synuclein pre-formed fibrils (PFFs) injection, intranasal ferric ammonium citrate (FAC) administration or chemogenetic inhibition of nigral dopaminergic neurons to further investigate the relationship between the damage of dopaminergic neurons in the SNpc and olfactory dysfunction. We demonstrated that the impaired performance in olfactory discrimination test was correlated with the loss of nigral dopaminergic neurons and restored when neurodegeneration was fully recued. This study shows a direct relationship between nigral dopaminergic neurodegeneration and olfactory dysfunction, and reinforces the current understanding that olfactory dysfunction represents an early event in PD progression.

## Results

### Severer olfactory dysfunction is not associated with lesions in the OB of rotenone induced PD mice model

**Fig. 1:**
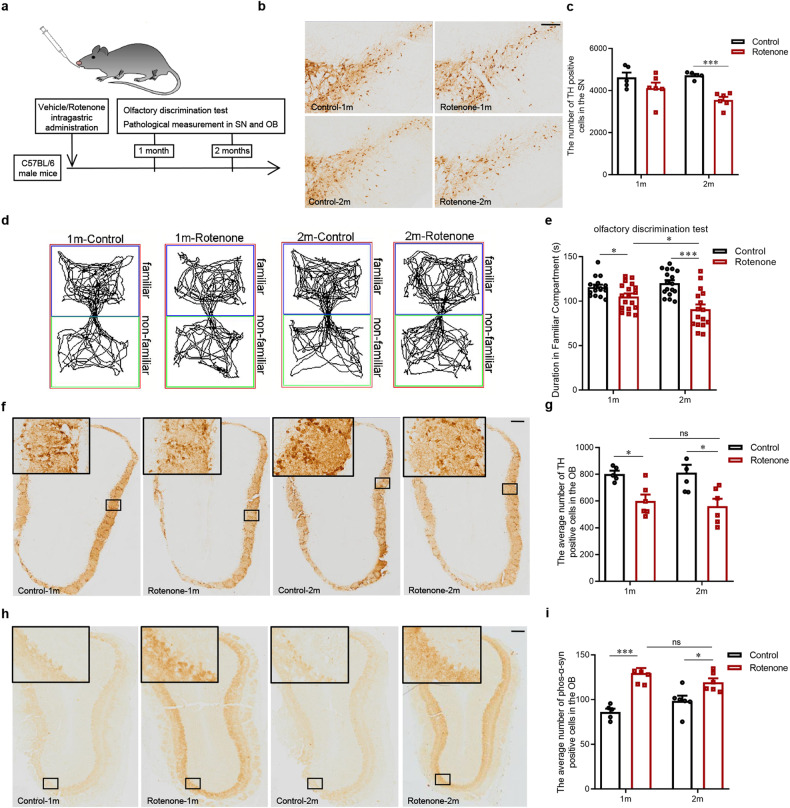
Olfactory dysfunction and dopaminergic neurodegeneration in the SNpc and OB of mice with rotenone administration. **a** Schematic model of intragastric administration of rotenone. Representative images of immunohistochemistry staining (**b**) and histogram of the number of TH positive cells (**c**) in the SNpc of mice (*n* = 5,6,5,6, ^***^P < 0.001). **d** Representative images of track visualization in the olfactory discrimination test. **e** Quantification of the accumulated duration in familiar compartments of mice (*n* = 16,18,17,16, ^*^P < 0.05, ^***^P < 0.001). Representative images of immunohistochemistry staining and histogram of the average number of TH (**f**, **g**) and phosphorylated α-synuclein positive cells (**h**, **i**) in the OB of mice (*n* = 5,6,6,6, ^*^P < 0.05, ^***^P < 0.001). Scale bar = 200 μm. Two-tailed Student’s *t* test was applied and data were presented as mean ± SEM.

Rotenone is a neurotoxin extensively used to model PD, as a common experimental model induced PD, replicating the degeneration of dopaminergic neurons in the SN [18]. Consistent with the previous publications [17, 19], we observed the loss of TH-immunopositive neurons in the SN of mice with intragastric rotenone administration for 2 months. However, no obvious neuronal loss was observed at 1 month after rotenone administration (Fig. 1a–c). We applied an olfactory discrimination test to evaluate the olfactory function in rotenone models. We observed the accumulated duration spent in familiar compartments decreased in mice with rotenone administration for 1 month indicating an impaired olfaction. Unexpectedly, a further reduction was observed in mice with rotenone administration for 2 months when compared to the 1-month group (Fig. 1d, e). In the rotenone models, the number of TH-immunopositive cells in the OB was decreased and meanwhile, the number of phosphorylated α-synuclein-immunopositive cells was increased, suggesting the lesion in the OB might be involved in the olfactory dysfunction. However, no differences were found in the rotenone-treated mice between 1 month and 2 months groups (Fig. 1f–i), suggesting that the damage of dopaminergic neurons and α-synuclein accumulation were not further aggravated. Therefore, we speculated severer olfactory dysfunction in rotenone models might not be associated with lesions in the OB.

### Nigral dopaminergic neurodegeneration is associated with olfactory dysfunction in mice model with intrastriatal α-synuclein PFFs injection

**Fig. 2:**
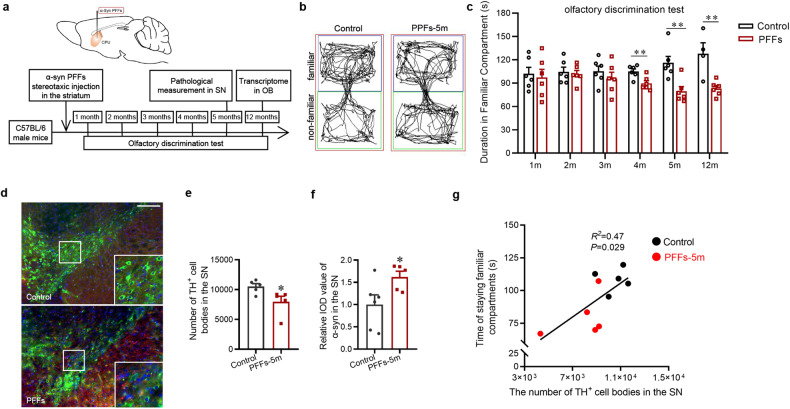
Olfactory dysfunction and nigral dopaminergic neurodegeneration in mice with α-synuclein PFFs injection. **a** Schematic model of stereotaxic injection of α-synuclein PFFs in the striatum. **b** Representative images of track visualization in the olfactory discrimination test. **c** Quantification of the accumulated duration in familiar compartments of mice at different timepoints (1-12 months) after α-synuclein PFFs intrastriatal injection (*n* = 6, ^**^P < 0.01) **d**–**f** Representative images and quantification of immunofluorescence staining of TH-positive cells (green, *n* = 5) and α-synuclein filaments (red, *n* = 6,5) and nucleus (DAPI) in the SN at 5 months after α-synuclein PFFs injection (^*^P < 0.05). Scale bar = 200 μm. **g** Pearson’s correlation analysis between the accumulated duration in familiar compartments and the number of TH-immunopostive cells in the SN at five months after α-synuclein PFFs injection. Two-tailed Student’s *t* test was applied and data were presented as mean ± SEM.

**Fig. 3:**
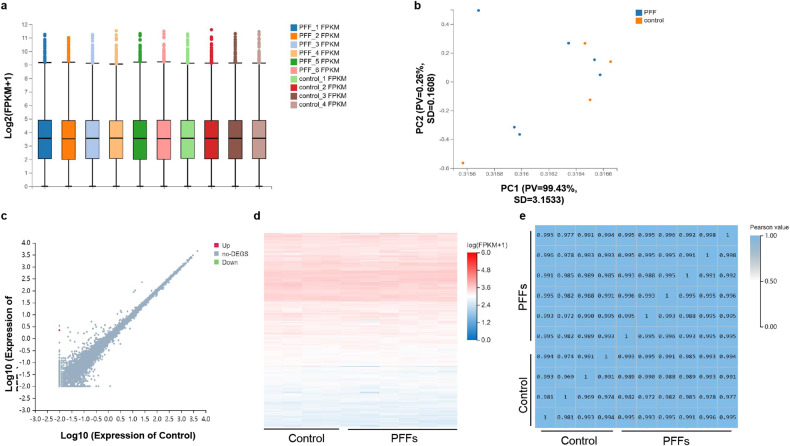
Transcriptome profiling of RNA-seq analyses in the OB of mice with α-synuclein PFFs injection. **a**, **b** Box plots of gene expression date and principal components analysis of OB tissues from the mice 12 months after intrastrital single injection of α-synuclein PFFs. **c**, **d** There is almost none differentially expressed gene in total of 18,623 genes detected (adjusted *P* < 0.05). **e** The correlation of expression profiles of individual OB tissues. Gene expressions are highly correlated. *n* = 4,6.

We then established mice model with a single injection of α-synuclein PFFs in the CPu [20, 21] (Fig. 2a). The olfactory discrimination test was applied at every month after α-synuclein PFFs injection. There was no difference in the accumulating duration spent in familiar compartments between α-synuclein PFFs and saline-injected groups at the first 3 months. A significant decrease of accumulating duration starts to appear at the fourth month and remains obvious at the twelfth month (Fig. 2b, c). At this point, as analyzed by transcriptome profiling in the OB, there were almost none differentially expressed gene in the OB virtually (adjusted *P* < 0.05, Fig. 3), suggesting almost nothing identified in the OB although there was a clear olfactory dysfunction. However, recognized by anti-α-synuclein filament antibody [MJFR-14-6-4-2]-Conformation-Specific, we observed that the integrated optical density (IOD) value of α-synuclein were increased definitely, indicated α-synuclein aggregates in the SNpc of mice 5 months after PFFs injection (Fig. 2d, f). Meanwhile, a 24.3% reduction in the number of TH-immunopositive cells was observed (Fig. 2d, e). More importantly, there was correlations between the accumulated duration spent in familiar compartments and the number of TH-immunopositive cells in the SNpc (Pearson’s correlation coefficient R^2^ = 0.47, *p* = 0.029, Fig. 2g), suggesting nigral dopaminergic neurodegeneration is associated with olfactory dysfunction in intrastriatal PFFs-inoculated mice.

### Nigral dopaminergic neurodegeneration is associated with olfactory dysfunction in mice model with intranasal FAC administration

**Fig. 4:**
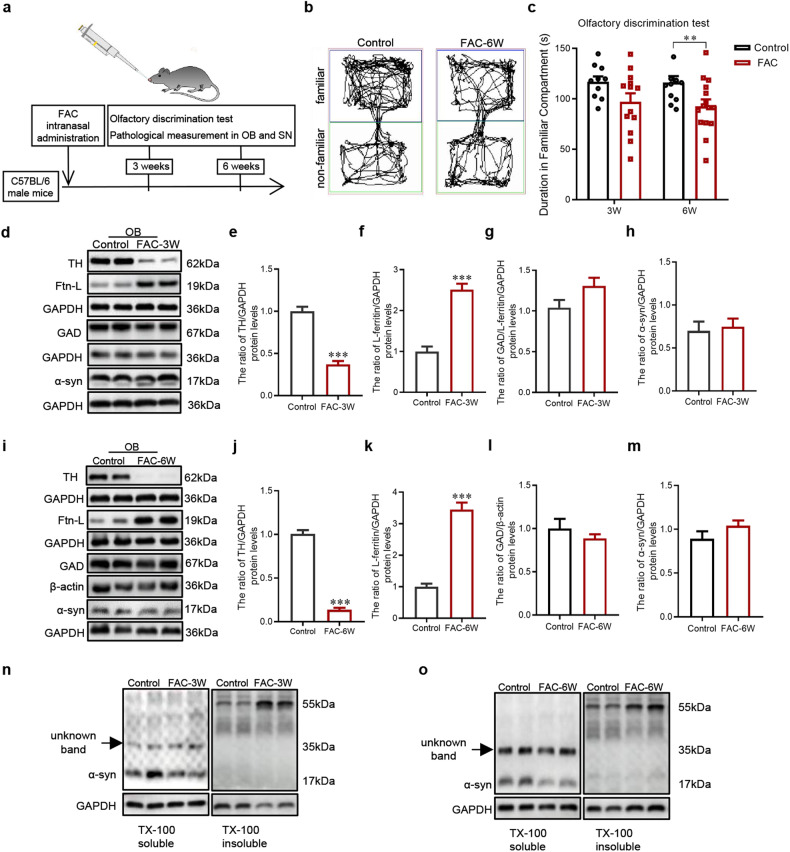
Evaluation of olfactory discrimination and OB lesions in mice with FAC administration for 3 weeks or 6 weeks. **a** Schematic model of intranasal FAC administration. **b** Representative images of track visualization in the olfactory discrimination test. **c** Quantification of the accumulated duration in familiar compartments of mice (*n* = 10,13,12,15, ^*^P < 0.05). **d**–**h** Western blots were used to detect the protein levels of TH (*n* = 5), L-ferritin (*n* = 5), GAD (*n* = 5,8) and α-synuclein (*n* = 5) in the OB with FAC administration for 3 weeks (^***^P < 0.001^)^. **i**–**m** Western blots were used to detect the protein levels of TH (*n* = 6), L-ferritin (*n* = 6), GAD (*n* = 5,6) and α-synuclein (*n* = 6) in the OB with FAC administration for 6 weeks (^***^P < 0.001). **n, o** Western blots were used to detect the protein levels of α-syn in soluble and insoluble fractions in the OB with FAC administration for 3 weeks or 6 weeks. Two-tailed Student’s *t-*test was applied and data were presented as mean ± SEM.

We further established mice models with intranasal FAC administration to investigate whether olfactory dysfunction is related to neurodegeneration in the SN (Fig. 4a). In mice with FAC administration for 3 weeks, we have not seen differences in the accumulated duration spent in familiar compartments between FAC and saline-administrated groups (Fig. 4b, c), indicating no significant alteration of olfaction at this time. The protein levels of light chain ferritin (L-ferritin) were upregulated by 151% in FAC group compared to the saline group, demonstrating iron deposits in the OB. Accordingly, the protein TH (marker for dopaminergic neurons), rather than GAD (marker for GABAergic neurons) (Fig. 4d–g), robustly decreased 61%, possibly due to the vulnerability of dopaminergic neurons to iron. Elevated insoluble α-synuclein indicated the existence of α-synuclein aggregation (Fig. 4m), although we did not observe the alterations of total α-synuclein levels (Fig. 4h). Despite of the obvious lesion in the OB, there is no nigral iron deposition yet, as indicated by the results that protein levels of L-ferritin (Fig. S1a, b) and the number of iron-positive cells (Fig. S1c, d) are unchanged in the SN. Accordingly, the number of TH-immunopositive cells in the SNpc is comparable to the saline-administrated group (Fig. S1e, f), indicating no neurodegeneration in the SN occurs.

**Fig. 5:**
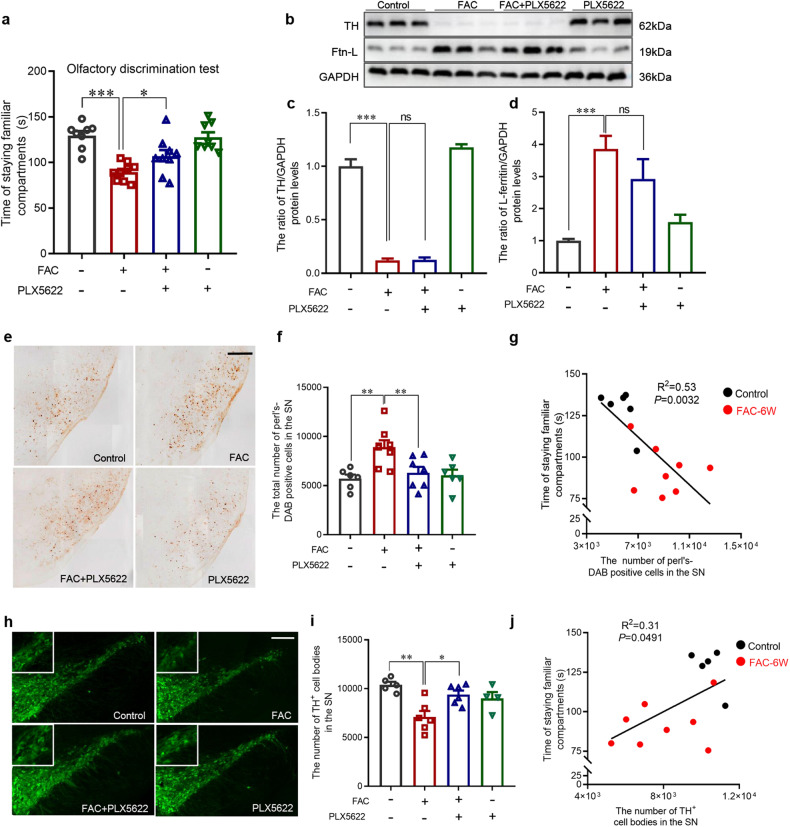
Effects of PLX5622-formulated diet on olfactory discrimination, OB lesions and SN lesions in mice with FAC administration. **a** Quantification of the accumulated duration in familiar compartments to evaluate the olfactory discrimination of mice (*n* = 8,9,9,8, ^*^P < 0.05, ^***^P < 0.001). Western blots were used to detect the protein levels of TH (**b**, **c**) and L-ferritin (**b**, **d**) in the OB (n = 6, ^***^P < 0.001). Representative images (**e**) and quantification (**f**) of Perl’s-DAB staining of iron positive cells in the SN of mice (*n* = 6,8,7,6, ^**^P < 0.01). **g** Pearson’s correlation analysis between the accumulated duration in familiar compartments and the number of Perl’s-DAB positive cells in the SN of mice with intranasal FAC administration for 6 weeks. Representative images (**h**) and quantification (**i**) of immunofluorescence staining of TH-positive cells (green) in the SN of mice (*n* = 5,6,6,4, ^*^P < 0.05, ^**^P < 0.01). **j** Pearson’s correlation analysis between the accumulated duration in familiar compartments and the TH-immunopositive cells in the SN of mice with intranasal FAC administration for 6 weeks. Scale bar = 200 μm. One-way ANOVA with Newman-Keuls multiple-comparison test were applied and data were presented as mean ± SEM.

Intranasal FAC administration was then applied for 6 weeks and more iron deposits in the OB as expected. A robust up-regulation of L-ferritin (245%) and a sever dopaminergic neurons loss (85%) (Fig. 4h–k), as well as the elevation of insoluble α-synuclein (Fig. 4n) were observed in the OB, although total α-synuclein levels remained unchanged (Fig. 4l). Meanwhile, mice with FAC, rather than saline administration exhibit an obvious decrease of accumulating duration in familiar compartments, indicating an impaired olfaction (Fig. 5a). A visible increase in the number of iron-positive cells by the Perl’s-DAB staining (Fig. 5e, f) and decrease in the number of TH-immunopositive cells (Fig. 5h, i) were observed in the SN, suggested an apparent iron deposition and dopaminergic neurodegeneration of these mice. Interestingly, Pearson’s correlation coefficients revealed a negative correlation between the accumulated time spent in familiar compartments and the number of Perl’s-DAB positive cells (Fig. 5g), as well as a positive correlation between the accumulated time spent in familiar compartments and TH-immunopostive cell bodies in the SN (Fig. 5j). These results suggested nigral dopaminergic neurodegeneration is associated with olfactory dysfunction in intranasal FAC administrated mice.

### Rescuing nigral dopaminergic neurodegeneration restores olfactory function in mice with PLX5622-formulated diet for 6 weeks

To further demonstrate the association between nigral dopaminergic neurons and olfaction, we tried to figure out whether the olfactory dysfunction could be restored when dopaminergic neurons are rescued. PLX5622-formulated diet was applied 3 days prior to intranasal FAC administration and continued for 6 weeks. PLX5622 is reported to ablate 99% microglia by pharmacologically inhibiting colony-stimulating factor 1 receptor [22]. As expected, nigral iron deposition (Fig. 5e, f) and the loss of dopaminergic neurons was fully blocked (Fig. 5h, i). We found the olfactory function was restored in these mice (Fig. 5a), however, there has been no amelioration in terms of the regulation of L-ferritin and TH in the OB induced by FAC treatment, indicating that PLX5622 did not exert any blocking effects of iron deposition and the loss of dopaminergic neurons in the OB (Fig. 5b–d). That means, the mice exhibit almost normal performance in the olfactory discrimination test, which is ascribable to the maintenance of nigral dopaminergic neurons.

### Chemogenetic inhibition of nigral dopaminergic neurons resulted in olfactory dysfunction

**Fig. 6:**
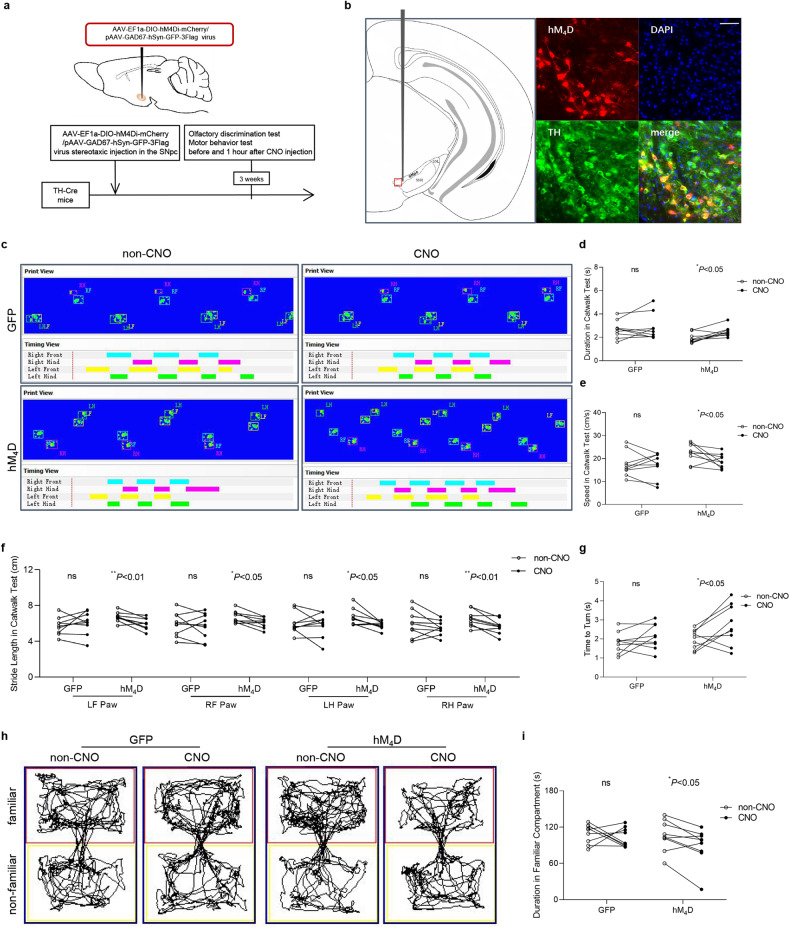
Motor deficits and olfactory dysfunction in mice with transient chemogenetic inhibition of dopaminergic neurons in the SN. **a** Schematic model of stereotaxic injection of virus in the SN. **b** Representative images of immunofluorescence staining of TH-positive cells (green) and AAV-EF1a-DIO-hM4Di-mCherry (hM_4_D) virus (red) (bar = 50 μm). **c** Representative images of paws’ print visualization in the Catwalk test. Duration (**d**) and speed (**e**) of mice crossing the monitoring area, stride length of all four paws (**f**) in the Catwalk test. (LF left front, LH left hind, RF right front, RH right hind). **g** Time to turn in the pole test. Representative images of track visualization in the olfactory discrimination test (**h**) and quantification of the accumulated duration in familiar compartments (**i**) of mice. (*n* = 9, ^*^P < 0.05, ^**^P < 0.01). Paired *t* test was applied.

To observe the effect of transient chemogenetic inhibition of dopaminergic neurons in the SN on olfaction, we established mice model with bilateral injection of hM_4_D or GFP virus in the SN (Fig. 6a). The virus was successfully fused with dopaminergic neurons in the SNpc (Fig. 6b). One hour after intraperitoneal CNO injection, the mice showed elevated run duration (Fig. 6d) and decreased run speed (Fig. 6e) in the Catwalk test; meanwhile, the decreased stride length of all four paws (Fig. 6c, f) were observed. In the pole test, time to turn was prolonged (Fig. 6g). The above suggested that the inactivation of dopaminergic neurons caused motor deficits. As we expected, olfactory discrimination test results showed that, the accumulated time spent in familiar compartments was longer after the intraperitoneal injection of CNO (2 mg/kg) compared to that before injection (Fig. 6h, i). None of the above changes was observed in the GFP group mice with the control virus administration, which ruled out the effect of CNO.

## Discussion

In this study, we first used mice model with intragastric rotenone administration and demonstrated that further impairment of olfactory function wasn’t accompanied by further damage in the OB, however, associated with the loss of dopaminergic neurons in the SNpc. The relationships between olfactory function and nigral dopaminergic neurons was further supported by the data from mice models with intrastriatal α-synuclein PFFs injection, intranasal FAC administration or chemogenetic inhibition of nigral dopaminergic neurons. A neuroanatomical basis from the SNpc to OB was demonstrated in rats via axonal tracing and 6-hydroxydopamine, which destroyed nigral dopaminergic neurons, reduced these dopaminergic projections [23], our study provides functional lines of evidence that dopaminergic neurodegeneration in the SNpc is associated with olfactory dysfunction in PD models.

OB is supposed to be early involved in α-synuclein pathology in Braak Staging, which is usually believed to be associated the early occurrence of non-motor symptom (hyposmia) before the onset of motor symptom in PD [6]. The olfactory vector hypothesis is supported in this scenario, by the evidence from both PD cases and mice models. A lower global glomerular voxel volume is defined in the OB and correlates with high phosphorylated α-synuclein immunoreactivity in PD cases [24]. In the mice with local α-synuclein overexpression in the OB, olfactory perception is impaired as indicated by the buried food pellet test and olfactory preference/avoidance test [10]. Consistent with these previous observations, our study implicates that impaired olfactory discrimination and phosphorylated α-synuclein immunoreactivity in the OB of rotenone induced PD models.

In the present study, we also observed the loss of dopaminergic neurons in the OB of rotenone induced PD models. As for dopaminergic neurons in the OB, it was reported that local 6-hydroxydopamine injection in the OB was able to decrease the number of dopaminergic neurons and produce olfactory impairment in rodents, suggesting that the latter is likely linked to the loss of dopaminergic neurons [25]. However, there is evidence that PD patients showed a 100% increase in dopaminergic neurons in the OB, and the authors claimed hyposmia might be due to the increase of dopaminergic neurons [26]. Another study revealed PD subjects had no significant changes in dopaminergic neurons in glomerular layer, although fewer mitral/tufted neurons and calretinin expressing interneurons [27]. Upon these inconsistent data, we suppose it is hard to conclude whether the number of dopaminergic neurons in the OB is associated with olfactory function. Further evidence was demonstrated in the present study that in mice with intranasal FAC administration for 3 weeks, an obvious down-regulation of TH protein levels was observed, however, no alteration was observed in olfactory discrimination test of these mice. Therefore, olfactory functions in rotenone models and iron-overload models are not necessarily dependent on dopaminergic neurons in the OB.

Of note, we observed a much severer impairment in olfactory discrimination test in mice with rotenone administration for 2 months when compared to those for 1 month. However, the loss of dopaminergic neurons and α-synuclein pathology (accumulation of phos-α-synuclein) were not aggravated in the OB. Therefore, we speculate that the further deterioration in olfactory discrimination test in rotenone models might not be due to the further pathological alterations in the OB. Consistent with the previous reports that rotenone is able to cause dopaminergic neurodegeneration in the SN [15, 28], we observed the number of dopaminergic neurons in the SNpc began to decrease at the timepoint of 2 months. Therefore, we hypothesized the loss of nigral dopaminergic neurons might be associated with olfactory functions based on the dopaminergic projections from the SN to OB [23]. As the current clinical studies showed, reduced dopamine transporter uptake of the bilateral caudates was observed in hyposmic PD compared with the normosmic PD patients [29], although another study showed olfactory dysfunction in early PD is not correlated with dopamine uptake (as a measure for dopaminergic degeneration), but with dopamine turnover (as a marker for dopaminergic presynaptic compensatory processes) [30]. A more recent study reported olfactory dysfunction was correlated with striatal dopamine uptake only in the tremor-dominant subtype [31], suggesting the relationship between olfactory dysfunction and dopaminergic degeneration might be affected by PD subtypes. We then established the mice models with intrastriatal α-synuclein PFFs injection, and we observed an impaired olfactory discrimination at 4–12 months after the single injection. Alpha-synuclein PFFs has been widely used for investigating the intercellular transmission of the pathological protein [20, 32, 33]. As expected, α-synuclein aggregates are observed in the dopaminergic neurons of SNpc, as well as loss of of dopaminergic neurons in the SNpc. The correlation between the performance in olfactory discrimination test and the number of nigral dopaminergic neurons is analyzed, meanwhile, as analyzed by transcriptome profiling in the OB, there were almost no differentially expressed gene in the OB virtually. These data provide strong evidence that dopaminergic neurodegeneration in the SNpc is associated with olfactory dysfunction.

Intranasally administrated iron might deposit first in the olfactory epithelium and translocate to the OB via the axons of the olfactory sensory neurons [34]. Decreased TH levels, rather than GAD levels, indicated the loss of dopaminergic neurons in the OB of mice with FAC administration for 3 weeks, further supporting the fact that dopaminergic neuron is particularly susceptible to iron [35]. Iron deposition and dopaminergic neurodegeneration were much severer in the OB of mice with intranasal FAC administration for 6 weeks, which is reasonable considering iron definitely translocated more to the OB with longer exposure. However, the total α-synuclein levels, as well as the α-synuclein pathology (as indicated by Triton-insoluble α-synuclein levels) are comparable between 3 weeks and 6 weeks. Meanwhile, we identified an apparent hyposmia in these mice, and we then ask the question that whether FAC administration for 6 weeks is sufficient to induce dopaminergic neurodegeneration in the SN. As we expected, the number of dopaminergic neurons in the SNpc in mice with FAC administration for 3 weeks was unchanged, indicating no neurodegeneration in the SN occurs at this time. Mice with FAC administration for 6 weeks exhibit apparent iron deposition and dopaminergic neurodegeneration in the SN. Moreover, olfactory dysfunction was fully rescued by the PLX5622 administration, which also fully restored the number of dopaminergic neurons in the SNpc. However, there is still severe iron deposition and dopaminergic neurodegeneration in the OB. Namely, the olfactory performance in discrimination test is impaired when SN damage occurred, and is restored when SN damage is rescued. More strikingly, Pearson’s correlation coefficients revealed olfactory discrimination correlates well with dopaminergic neurodegeneration in the SN of mice. This reinforces the idea that in mice with intranasal administration of FAC, dopaminergic neurodegeneration in the SN is associated with olfactory dysfunction.

We notice that in a novel α-synuclein BAC transgenic mice model of prodromal PD, the mice showed hyposmia at 9 months of age, and the authors interpreted the data as consistent with the aggregation of α-synuclein in the OB [36]. However, there is an apparent loss of TH-immunopositive cell bodies in the SNpc of these mice at 9 months of age. Actually, triton-soluble truncated α-synuclein levels in the OB were already increased at 3 months of age, which seems not sufficient to cause olfactory dysfunction at that time. So one could not exclude the possibility olfactory dysfunction is associated with the loss of dopaminergic neurons in the SN, even in the presence of α-synuclein pathology in the OB, considering the α-synuclein pathology might be extensive/unspecific in certain regions in such a model with genetic manipulations. We then applied chemogenetic approaches to investigate the direct relationship between the function of nigral dopaminergic neurons and olfaction. The data demonstrated that hyposmia appeared when CNO was injection to induce transient inactivation of dopaminergic neurons. Motor deficits were observed concurrently, strongly supported the idea that dopaminergic neurodegeneration in the SNpc is associated with olfactory dysfunction in PD.

There are complex neural projection links between the OB and other brain regions, these projections constitute a complicated network of olfactory information processing [37, 38], although the regions/lesions responsible for hyposmia are not fully specified yet. Actually, once hyposmia occurred in PD progression, no studies have shown that it can be restored, even with the levodopa therapy [26]. While loss of smell recovered fast under other conditions such as Corona Virus Disease 2019 [39]. Despite of the differential regions associated Lewy bodies pathology and conceptual progression in PD, dopaminergic neurons in the SN are inevitably the most affected parts [40]. In the present study, we put forward the understandings in our models that dopaminergic neurodegeneration in the SN is associated with olfactory dysfunction in PD, the latter as the prodromal symptom indicating the early stage of neurodegeneration. Considering that 50–80% of dopaminergic cells are already lost when motor symptoms occurred, olfactory dysfunction, as the prodromal symptom indicating the early stage of nigral dopaminergic neurodegeneration, representing a slight perturbation in the SN possibly, is worthy of more emphasis in diagnosis and therapeutical studies of PD.

## Materials and methods

### Animals

All procedures to maintain and use mice were in accordance with the NIH Guide for the Care and Use of Laboratory Animals and were approved by the Institutional Animal Care and Use Committee at the Qingdao University (QDU-AEC-2021334). All mice were maintained in standard conditions of controlled room temperature and humidity under12 h light/dark cycle and free access to water and food. Mice were randomly assigned to different experimental groups. Sample sizes were based in standard protocols in the field and no sample or animal was excluded.

Eight-month-old C57BL/6 J male mice from Beijing Vital River Laboratory Animal Technology Co., Ltd (China) and maintained until twelve-month-old. These mice were administered orally once daily (5 days per week) at a dose of 6.25 mg/kg/d rotenone for 1 month and 2 months. Rotenone solution was prepared as follows. 0.72 g NaCl and 3.2 g Carboxymethylcellulose sodium were added to 79 ml ddH_2_O for full dissolution, followed by 1 ml chloroform pre-dissolved 50 mg rotenone, and then thoroughly mixed. Twelve-month-old C57BL/6 J male mice in the control group received only the vehicle without rotenone.

Eight-week-old C57BL/6 J male mice from GemPharmatech (China) weighing 19–23 g were also maintained in standard conditions described above. For intracerebral injection experiments, mice were applied with a single injection of 5 μg α-synuclein PFFs into the caudate putamen (CPu) and the control mice were applied with a single injection of saline accordingly. For intranasal administration experiments, mice were randomly picked and administrated with FAC (Sigma, USA) with a dosage of 200 mg/kg for three or six weeks, and the control mice were intranasally administrated with saline accordingly. For colony-stimulating factor 1 receptor inhibitor PLX5622 (Med Chem Express, USA) diet, 1200 PPM was added to normal chow. One week prior to FAC administration, PLX5622 diet was given and continued to apply in the period of FAC administration for 6 weeks.

Tyrosine hydroxylase (TH)-Cre transgenic mice (B6.Cg-7630403G23RikTg (Th-cre) 1Tmd/J) were obtained from The Jackson Laboratory (USA) and maintained on a C57BL/6 J background. For virus administration, male and female heterozygous mice, aged 8–20 weeks, were randomly picked and used.

### Assembly of α-synuclein monomers into fibrils

Alpha-syn PFFs were generated by incubating human recombinant α-syn (rPeptide, USA) to a 5 mg/mL solution (buffered with 50 mM Tris-HCl, 150 mM KCl, pH = 7.4) at 37 °C with constant shaking at 1000 rpm for 7 days. During the incubation, the solution becomes turbid and the typical filaments of the fibrils were observed with a transmission electron microscope. The α-syn PFFs were subpackaged, frozen at −80 °C and ultrasonicated in water bath with working fluid concentration for 1 min before injection.

### Stereotaxic surgery

For bilateral α-synuclein PFFs administration, anesthetized mice were placed into a stereotaxic device and either α-synuclein PFFs or sterile saline. The bilateral infusion of 1 μL of PFFs (5 μg/μL) or saline were injected into the CPu by electronic infusion pump at a rate of 0.1 μL/min for 10 min. Coordinates to bregma for injection were +0.4 mm (AP), ±1.8 mm (ML) and −3.5 mm (DV). For bilateral virus administration, 100 nL of AAV-EF1a-DIO-hM4Di-mCherry (hM_4_D, virus diluted at 1:2) was injected into the SN. Coordinates to bregma for injection were −2.92 mm (AP), ±1.15 mm (ML) and −4.64 mm (DV). Virus pAAV-GAD67-hSyn-GFP-3Flag (GFP, virus diluted at 1:2) was chosen as control vector. Vector and virus were purchased from OBiO Technology Company (Shanghai, China). The needle was left in place for 10 minutes after each injection before being removed. All animals were recovered on heating pads at 37 °C and monitored regularly.

### Olfactory discrimination test

Olfactory function was examined using the accumulated time spent in familiar compartments. The test was done in a transparent acrylic box (30 cm×20 cm×20 cm) with two compartments equally divided, and an arched passage for the mice to walk through freely. Before the test, the animals were habituated to the environment for 2 min, with clean paddings on one side of the box (non-familiar compartments), old paddings in which the mice remained live for 3–5d before testing on the other side of the box (familiar compartments). In the test, each mouse was placed in the middle of arched passage and recorded the accumulated time spent in each compartment for 3 min by the system of Ethvision XT7.

### Motor behavior tests

CatWalk XT gait analysis system (Noldus) was used to automatically record and analyze the gait pattern of the mice [41]. Mice were familiarized with the test environment one day before recording. In the recording, mice walked freely through the glass walkway and were captured through a high-speed camera under the walkway. Several static and dynamic parameters were analyzed to describe and assess the symptomatic motor deficits.

In the pole test [42], a metallic pole wrapped with medical tape with a diameter of 0.5 cm and height of 50 cm was placed vertically in a cage (40 cm × 60 cm) and covered with bedding in the cage to protect the mice from injury by falling. A tape covered rubber ball was glued on top of the pole. Mice were pre-trained three times to learn how to climb down one day before the formal test. Segmented timing the time to turn around and the time it took for the mice to get from the top to the bottom after turn. Three measurements were made and average values were taken for statistics. The duration, especially the time to turn, reflects the change of motor coordination in mice.

### Clozapine N-oxide (CNO) injection

Three weeks after virus injection with stereotaxic surgery (virus fused with neurons), 2 mg/kg CNO was injected intraperitoneally. Olfactory discrimination test and motor behavior tests were performed before injection and 30–120 min after injection respectively.

### Immunofluorescence staining

Mice were euthanized and perfused with saline and then ice-cold 4% paraformaldehyde (PFA) in 0.1 M phosphate buffered saline (PBS) for 12–24 h, then the brains were placed in 30% sucrose solution for 24 h and cut into 20 μm slices by machine of frozen slicer (Leica CM1950). For immunofluorescence, sections are fixed in 4% PFA for 10 min and rinsed in PBS for 10 min each time for three times. Subsequently, sections are permeabilized in 5% donkey serum (Jackson Immuno Research, USA) containing 0.3% Triton X-100 (Biosharp, South Korea) at room temperature and incubated at 4 °C overnight. TH primary antibody (1:1000, Abcam, UK) and anti-α-synuclein filament antibody [MJFR-14-6-4-2]-Conformation-Specific (1:5000 Abcam, UK) were diluted in 0.01 M PBST. The sections were incubated with a secondary antibody (1:500 Invitrogen, USA) for fluorescence staining at room temperature for 2 h. The sections were then mounted onto glycerin-coated slides and cover slipped. The images were obtained by a confocal microscope (Olympus VS120, Japanese) and quantifications were performed by the software OlyVIA\xvViewer and ImageJ.

### Perl’s- 3,3’-Diaminobenzidine (DAB) staining

Iron deposition was detected with Perl’s-DAB staining. Briefly, after fixation in 4% PFA, the sections were incubated for 30 min in a mixture containing 1% HCl-1% potassium ferrocyanide (sigma, USA) and then were rinsed in PBS. After that, the sections were incubated in 3% H_2_O_2_ of methanol solution for 20 min. After washing in PBS, the sections were incubated with DAB for 10 min and placed in ddH2O finally. The sections were then mounted onto gelatin containing xylene coated slides and cover slipped after dehydration. The images were obtained by a microscope (Olympus VS120, Japanese) and quantifications were performed by the software OlyVIA\xvViewer.

### Western Blot

The OB and SN were collected and lysed in were lysed with RIPA lysis buffer (Beyotime, China) and protease inhibitor (Roche, USA). The protein concentration was determined using the BCA protein assay kit (CWBIO, USA) and. The samples of 15 μg were electrophoresed and separated on 10% sodium dodecyl sulfate-polyacrylamide gel. The proteins were then transferred to polyvinylidene difluoride membrane (Millipore, USA) for 90 min. After blocking for 2 h with 5% non-fat milk in TBST at room temperature (RT), the membranes were incubated with primary antibodies against TH (1:3000, Millipore, USA), L-ferritin (1:1000, Abcam, UK), glutamic acid decarboxylase (GAD, 1:10000, Abcam, UK), GAPDH (1:20000, absin, China), β-actin (1:10000, Bioss, China) overnight at 4 °C. After washing in PBS, the membranes were incubated with horseradish peroxidase–conjugated secondary antibodies (1:10000, absin, China) were applied for 1 h at RT. Immunoreactive bands were detected and visualized with Fusion system (UVP, USA). The band intensities were normalized to those of β-actin or GAPDH.

### RNA-seq

RNA-sequencing is performed by Beijing Genomics Institute (BGI). Total RNA was extracted from the tissues using Trizol (Invitrogen, Carlsbad, CA, USA) according to manual instruction. Finally, 25 µL ~ 100 µL of DEPC-treated water was added to dissolve the RNA. Subsequently, total RNA was qualified and quantified using a Nano Drop and Agilent 2100 bioanalyzer (Thermo Fisher Scientific, MA, USA). Oligo(dT)-attached magnetic beads were used to purified mRNA. The cDNA fragments obtained from previous step were amplified by PCR, and products were purified by Ampure XP Beads, then dissolved in EB solution. The final library was amplified with phi29 to make DNA nanoball (DNB) which had more than 300 copies of one molecular, DNBs were loaded into the patterned nanoarray and single end 50 bases reads were generated on BGIseq500 platform (BGI-Shenzhen, China). A gene was considered to be differentially regulated when Q or *P* < 0.05.

### Statistical analysis

Data are presented as mean±S.E.M. Detailed *n* values for each panel in the figures were stated in the corresponding legends. All statistical analyses were performed by GraphPad Prism 8.0 Software. *P* values were calculated with a two tailed Student’s unpaired *t* test for two groups. One-way analysis of variance (ANOVA) with Newman–Keuls multiple-comparison test was used for comparisons within multiple groups. Paired *t*-test was used for comparisons before and after CNO injection. *P* values of <0.05 indicated statistically significant differences. The investigators were blinded to experiments and outcome assessment.

### Supplementary information

Supplementary materials

Original Data File

## Data Availability

All data that support the findings of this study are available upon reasonable request from the corresponding author. The RNA-seq datasets generated for this study can be found in the SRA metadata under accession PRJNA1001165.
