# Supplementary material for: Dopaminergic neurodegeneration in the substantia nigra is associated with olfactory dysfunction in mice models of Parkinson’s disease
Source: Cell Death Discov. 2023 Oct 21;9:388. doi: 10.1038/s41420-023-01684-8 (PMC10590405; doi:10.1038/s41420-023-01684-8)
Supplement: Supplementary file 1 — Supplementary materials [file 41420_2023_1684_MOESM1_ESM.docx]

**Figure and figure legend**


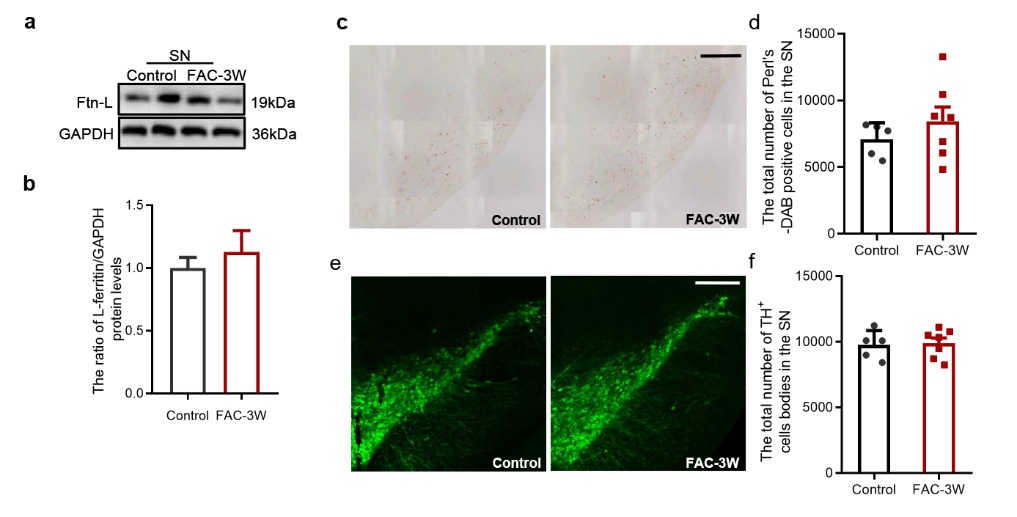


**Figure S1 No lesions in the SN of mice with FAC administration for 3 weeks. a-b** Western blot were used to detect the protein levels of L-ferritin in the SN (n=5). **c-f** Representative images and quantification of Perl’s-DAB staining of iron positive cells (c, d) and immunofluorescence staining of TH-positive cells (e, f) in the SN (n=5-7). Scale bar=200μm, Two-tailed Student’s t test was applied and data were presented as mean±SEM.
