# Supplementary figures and images for: Dopaminergic neurodegeneration in the substantia nigra is associated with olfactory dysfunction in mice models of Parkinson’s disease
Source: Cell Death Discov. 2023 Oct 21;9:388. doi: 10.1038/s41420-023-01684-8 (PMC10590405; doi:10.1038/s41420-023-01684-8)

**Raw image of western blots：**

**Fig. 4d FAC-3W-OB**


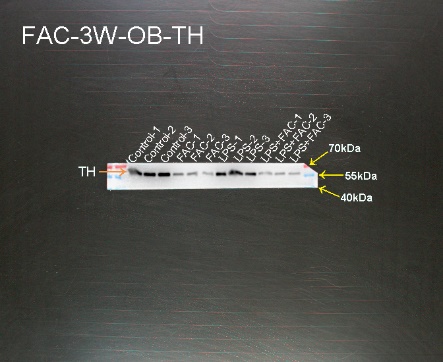

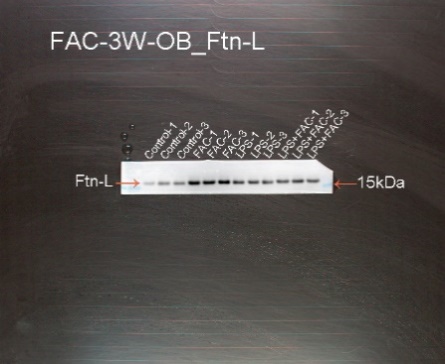

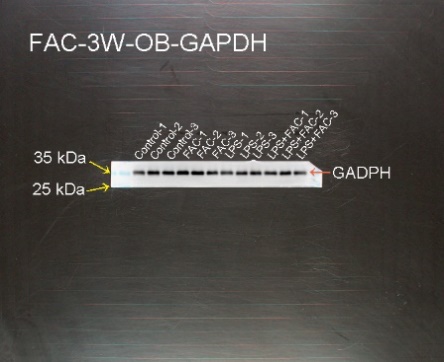


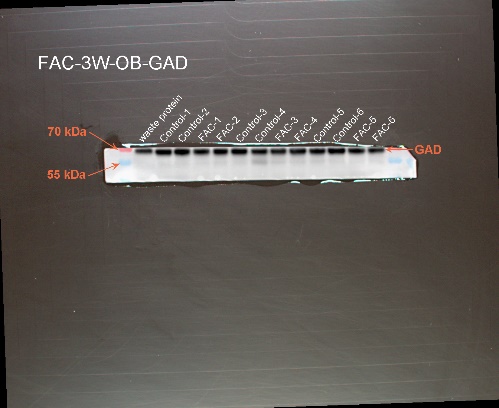

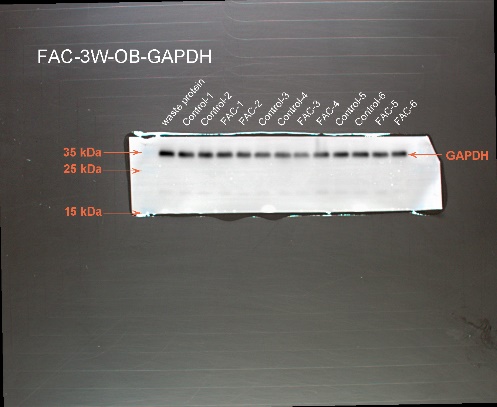




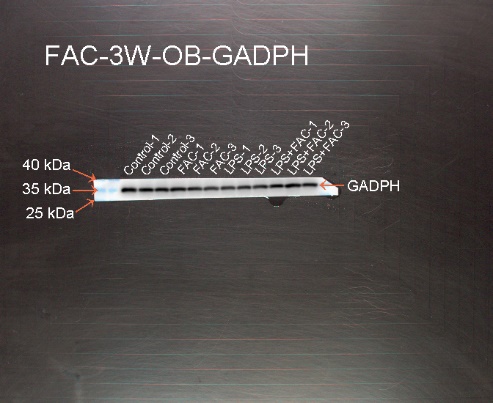


**Fig. 4i FAC-6W-OB**


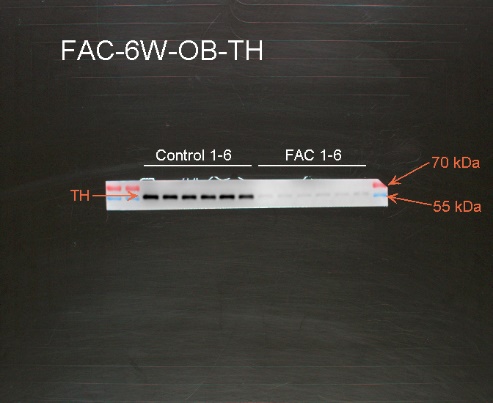

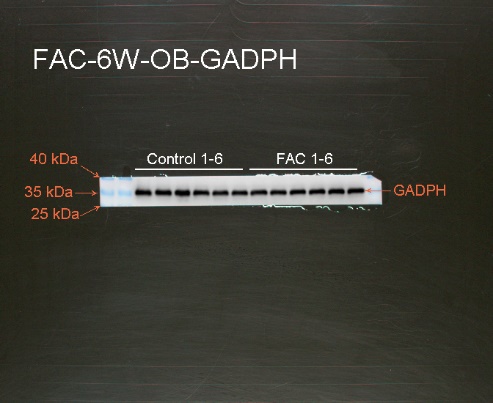


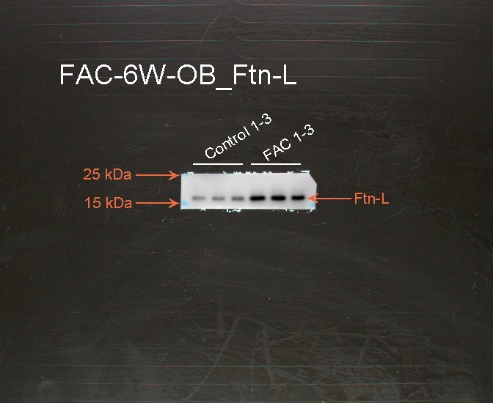

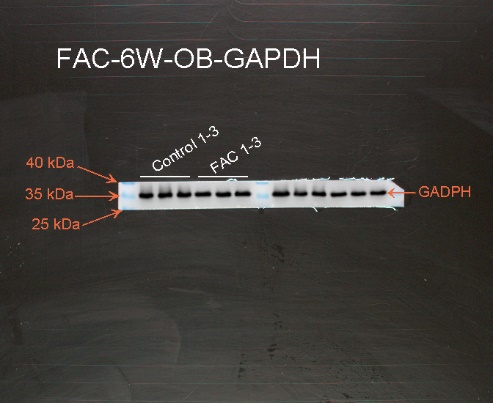


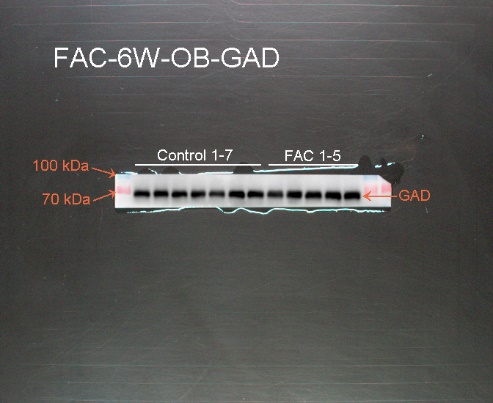

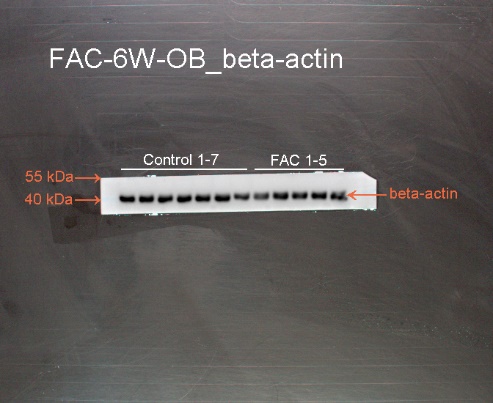


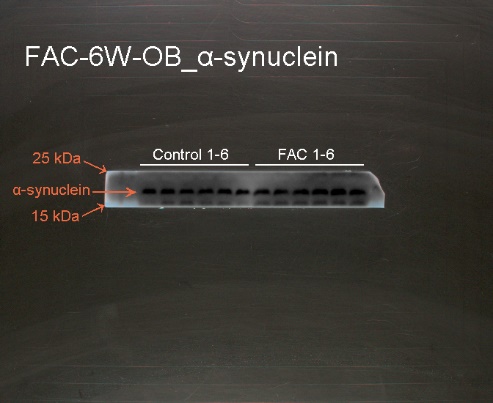

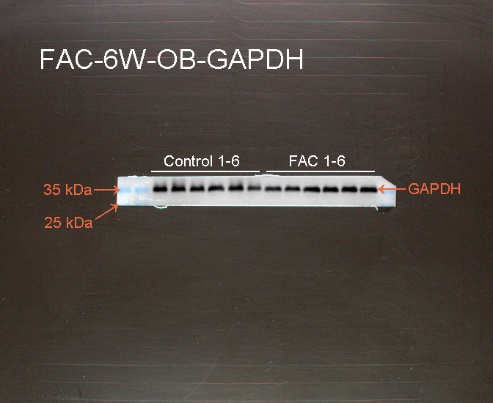


**Fig. 4n FAC-3W_TX-100**


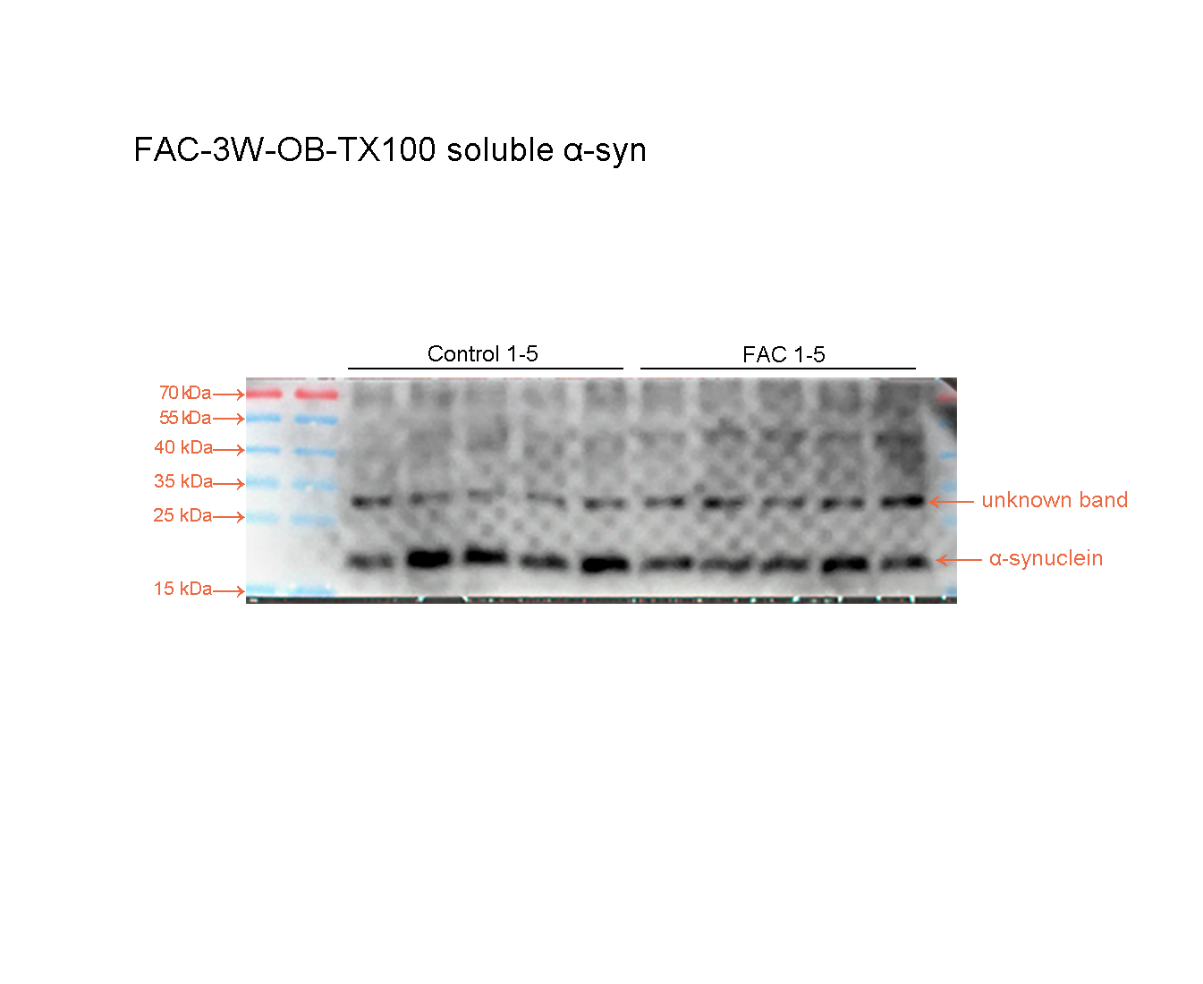

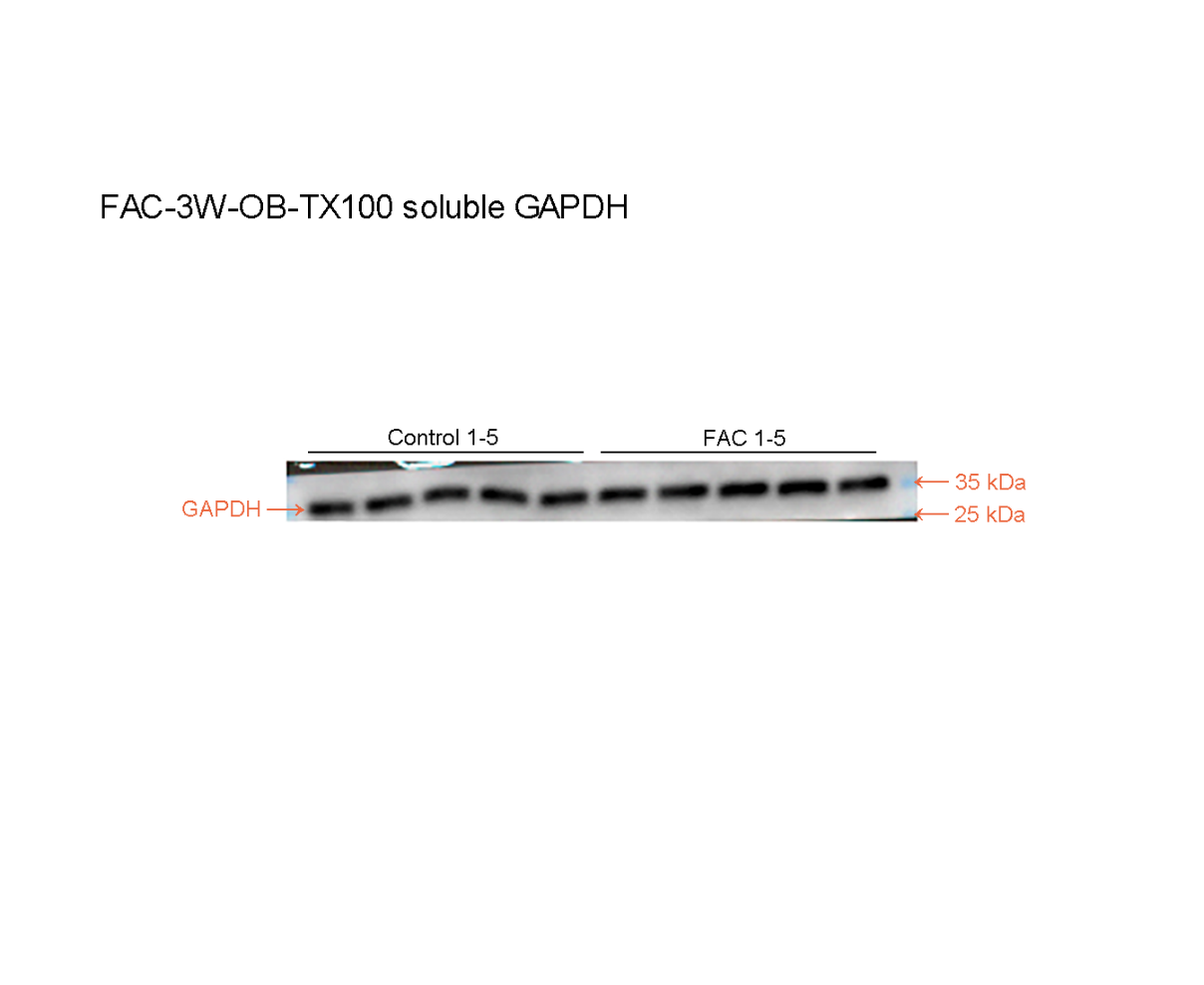

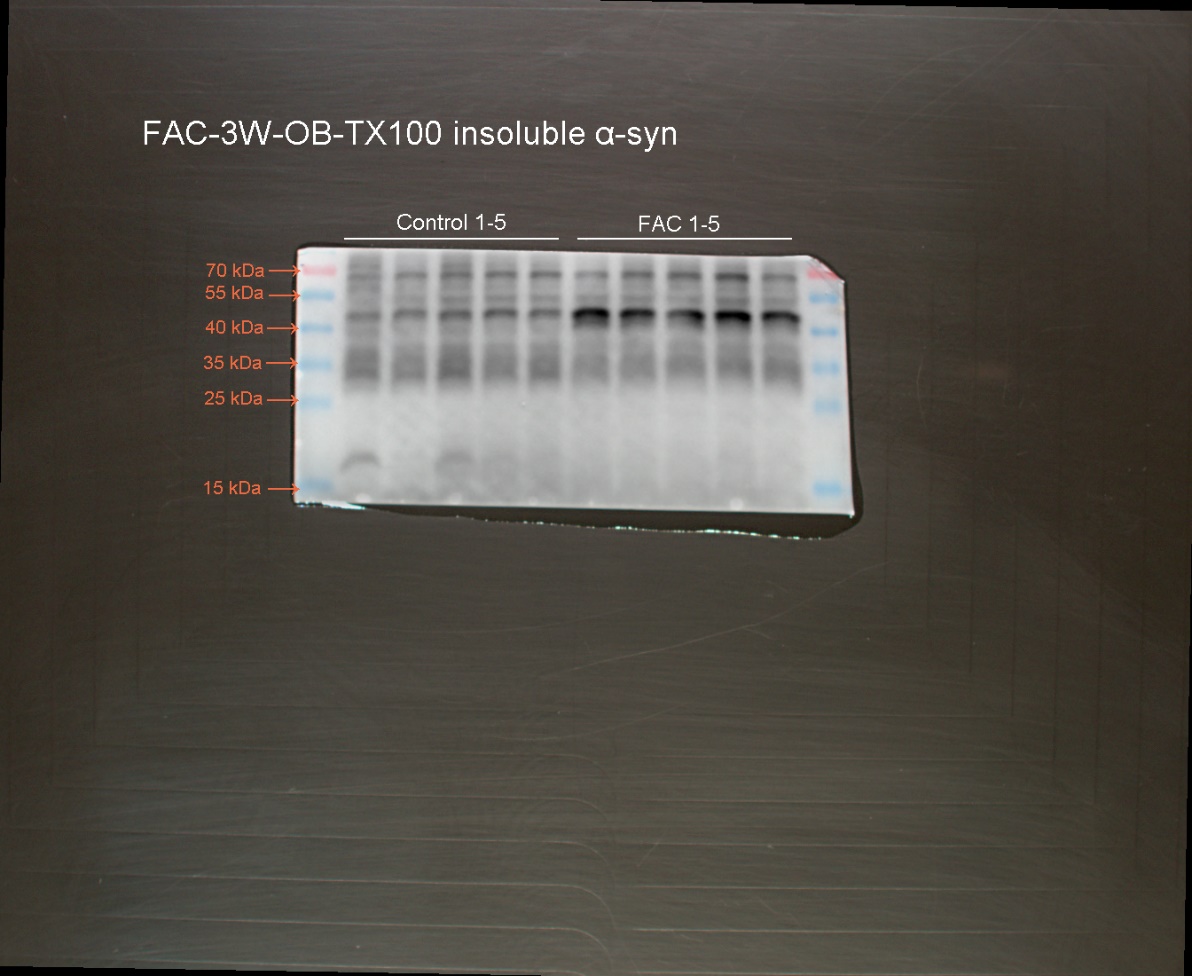

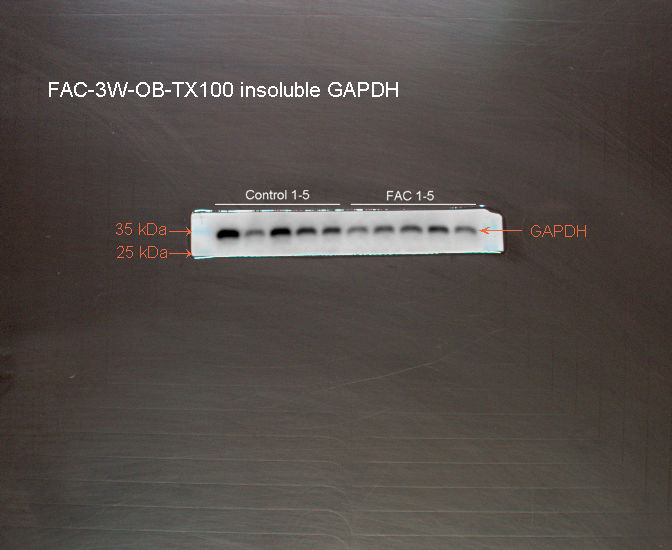


**Fig. 4o FAC-6W_TX-100**


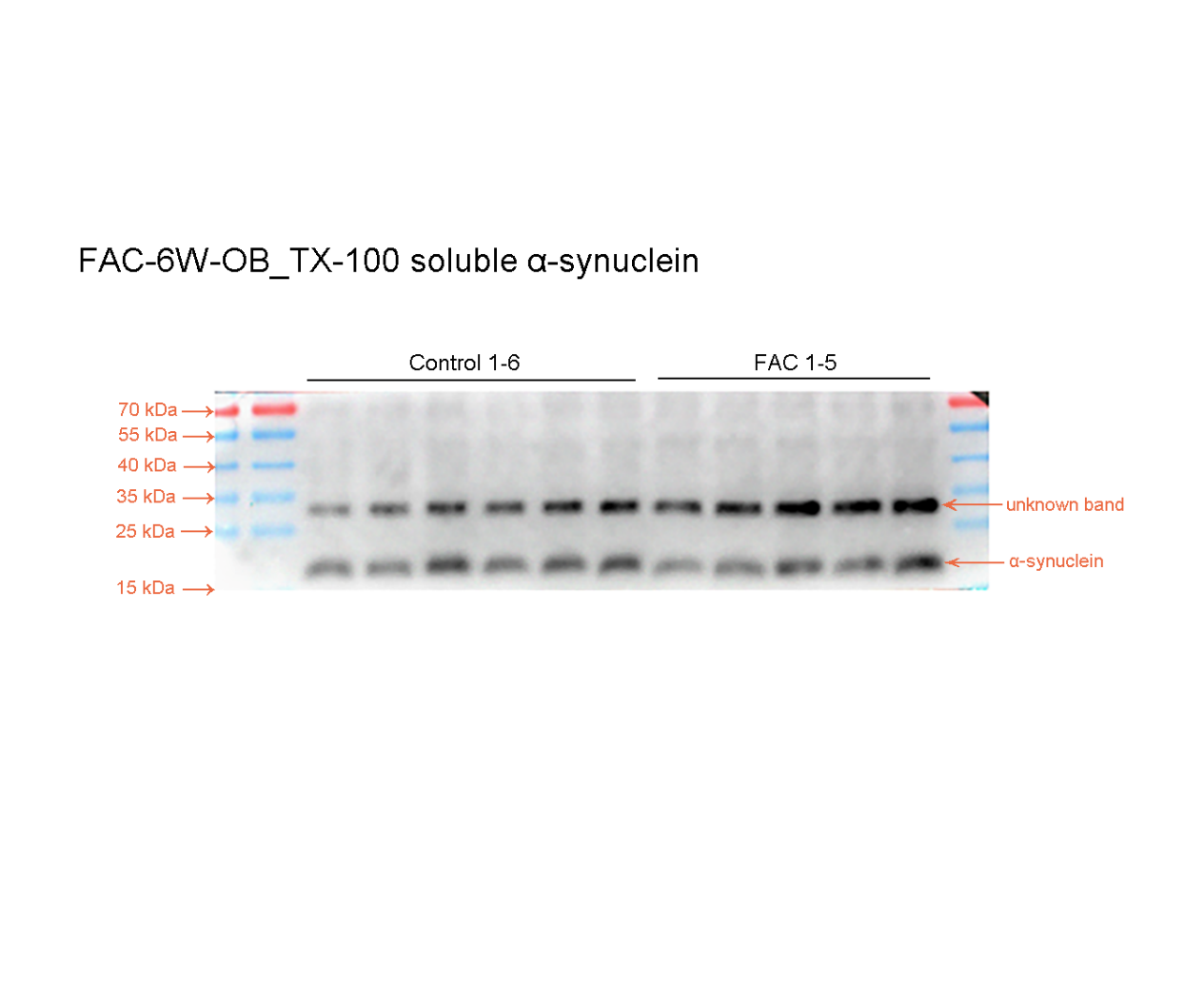

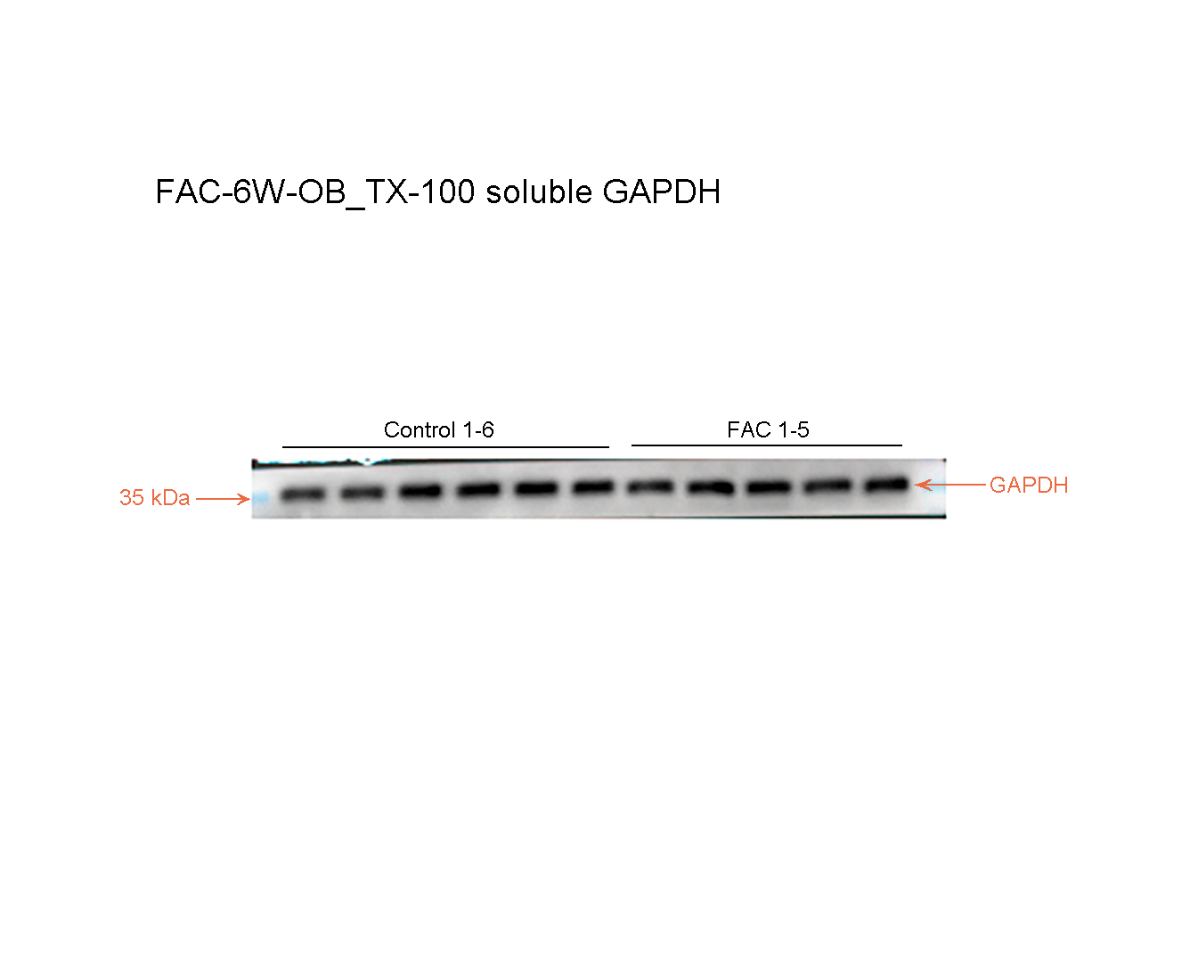

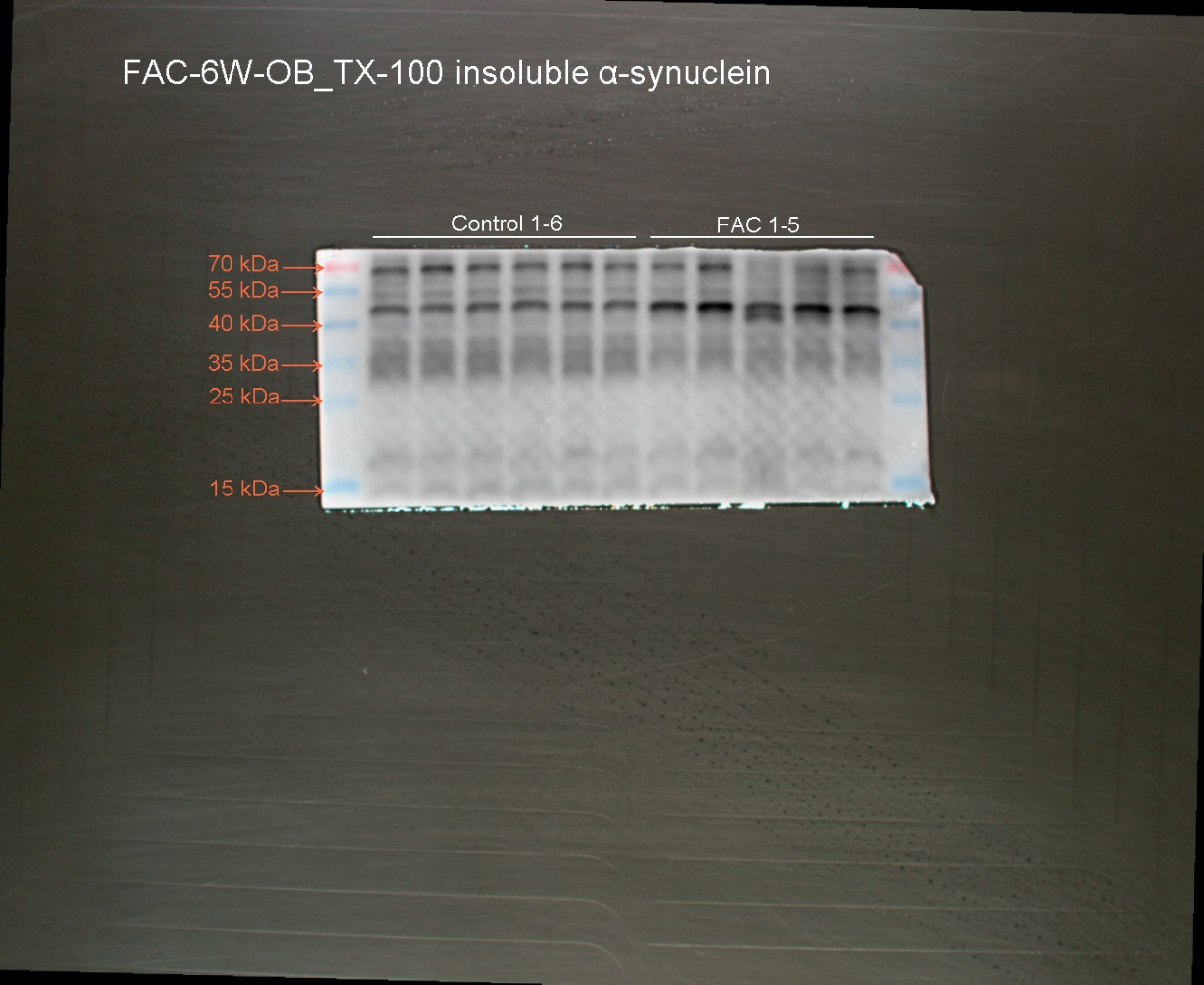

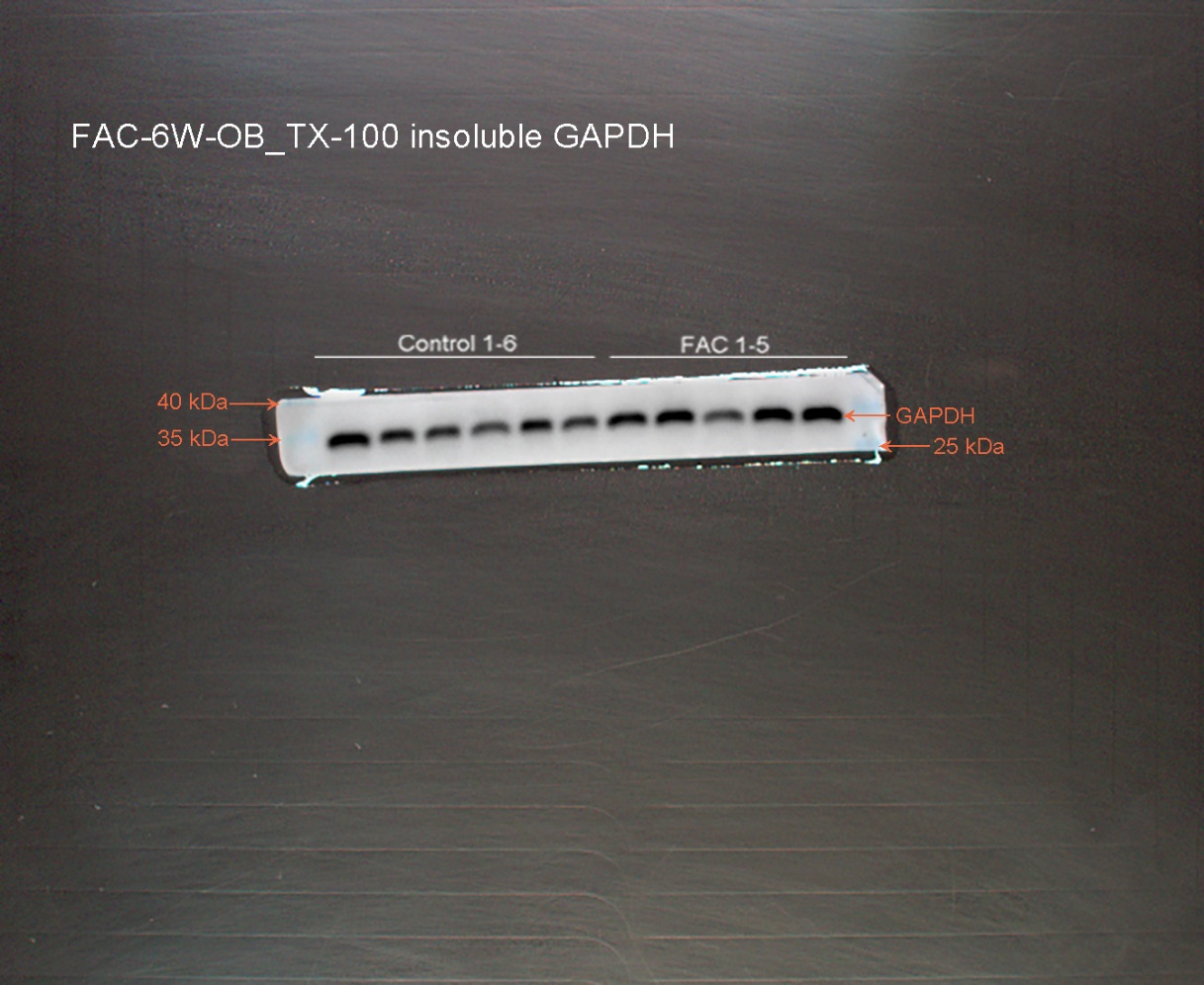


**Fig. 5b**


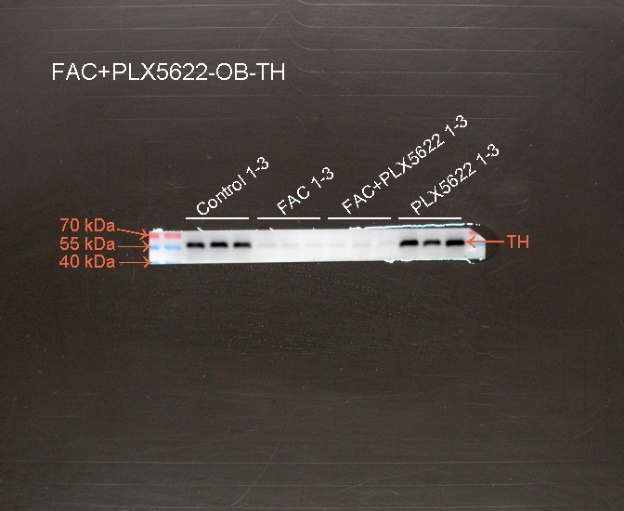

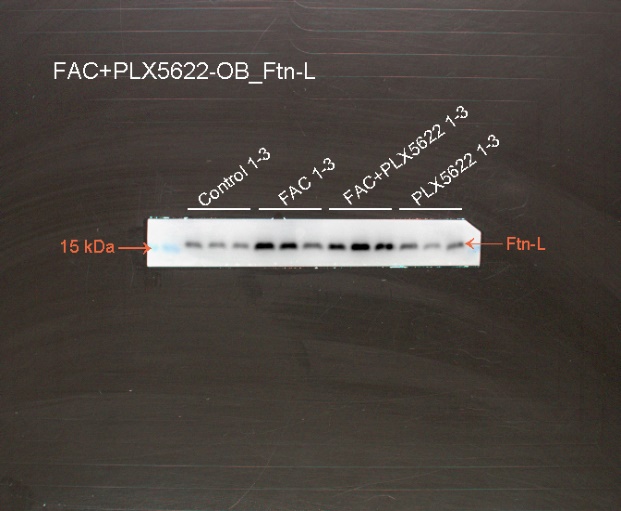


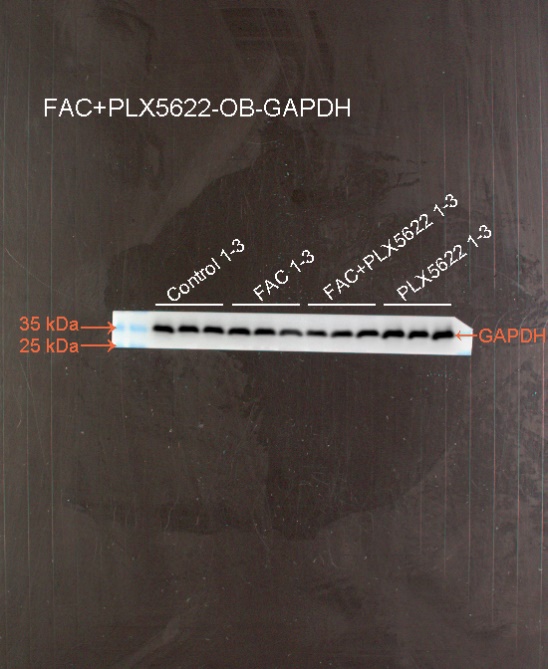


**Fig. S1a**


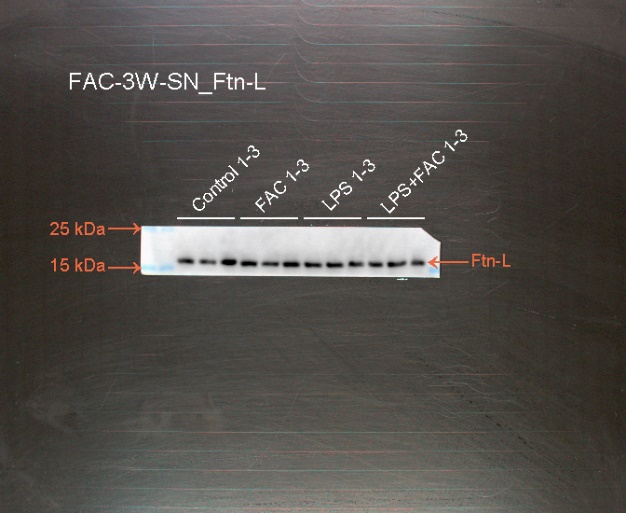

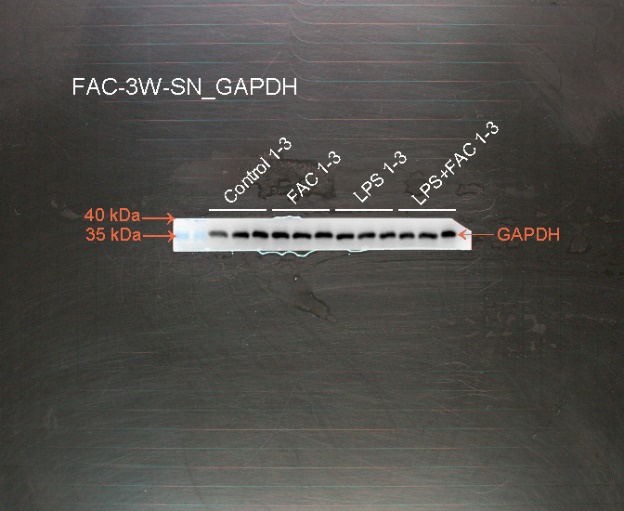

Supplement: Supplementary file 2 — Original Data File [file 41420_2023_1684_MOESM2_ESM.docx]
